# Usefulness of pyruvate dehydrogenase-E1α expression to determine SUVmax cut-off value of [^18^F]FDG-PET for predicting lymph node metastasis in lung cancer

**DOI:** 10.1038/s41598-023-28805-8

**Published:** 2023-01-28

**Authors:** Ryuichi Ito, Masakazu Yashiro, Takuma Tsukioka, Nobuhiro Izumi, Hiroaki Komatsu, Hidetoshi Inoue, Noritoshi Nishiyama

**Affiliations:** 1 Department of Thoracic Surgery, Osaka Metropolitan University, 1-4-3 Asahimachi, Abeno-ku, Osaka, 545-8585 Japan; 2 Molecular Oncology and Therapeutics, Osaka Metropolitan University Graduate School of Medicine, 1-4-3 Asahimachi, Abeno-ku, Osaka, 545-8585 Japan

**Keywords:** Cancer, Cancer imaging, Lung cancer, Tumour biomarkers

## Abstract

A more accurate cut-off value of maximum standardized uptake value (SUVmax) in [^18^F]fluorodeoxyglucose positron emission tomography/computed tomography ([^18^F]FDG-PET/CT) is necessary to improve preoperative nodal staging in patients with lung cancer. Overall, 223 patients with lung cancer who had undergone [^18^F]FDG-PET/CT within 2 months before surgery were enrolled. The expression of glucose transporter-1, pyruvate kinase-M2, pyruvate dehydrogenase-E1α (PDH-E1α), and carbonic anhydrase-9 was evaluated by immunohistochemistry. Clinicopathological background was retrospectively investigated. According to PDH-E1α expression in primary lesion, a significant difference (*p* = 0.021) in SUVmax of metastatic lymph nodes (3.0 with PDH-positive vs 4.5 with PDH-negative) was found, but not of other enzymes. When the cut-off value of SUVmax was set to 2.5, the sensitivity and specificity were 0.529 and 0.562, respectively, and the positive and negative predictive values were 0.505 and 0.586, respectively. However, when the cut-off value of SUVmax was set according to PDH-E1α expression (2.7 with PDH-positive and 3.2 with PDH-negative), the sensitivity and specificity were 0.441 and 0.868, respectively, and the positive and negative predictive values were 0.738 and 0.648, respectively. The SUVmax cut-off value for metastatic lymph nodes depends on PDH-E1α expression in primary lung cancer. The new SUVmax cut-off value according to PDH-E1α expression showed higher specificity for [^18^F]FDG-PET in the diagnosis of lymph node metastasis.

## Introduction

Lung cancer has high morbidity and mortality rates worldwide. Tumor-node metastasis (TNM) classification, particularly lymph node metastasis status, namely nodal stage, is important for determining the appropriate treatment^1^, such as the surgical procedure to be done and chemotherapy for patients with lung cancer^2,3^. The significance of various examinations, including computed tomography (CT), magnetic resonance imaging (MRI), endobronchial ultrasound-guided transbronchial needle aspiration (EBUS-TBNA), and [^18^F]fluorodeoxyglucose positron emission tomography/CT ([^18^F]FDG-PET/CT), has been evaluated for the diagnosis of clinical nodal stage. Based on superior results, [^18^F]FDG-PET/CT was found to be a useful tool for determining nodal stage in lung cancer^4^. In fact, before surgery, the appropriate region for lymph node dissection is mainly determined by [^18^F]FDG-PET/CT. However, surgical cases remain in which lymph node dissection is omitted due to negative [^18^F]FDG-PET/CT results, and metastatic lymph nodes are diagnosed by postoperative pathological examination^5^. If the accuracy of [^18^F]FDG-PET/CT can be improved to reduce the number of false-negative results, then more appropriate treatment strategies, such as chemotherapy and lymph node dissection, can be used. Thus, a more accurate method for identification of lymph node metastases using [^18^F]FDG-PET/CT is desired by thoracic surgeons^5,6^. Although the conventional cut-off for maximum standardized uptake value (SUVmax) of [^18^F]FDG-PET/CT to diagnose lymph node metastasis is ≥ 2.5^7–9^, a more accurate cut-off value is necessary to improve preoperative nodal staging.

The [^18^F]FDG-PET/CT imaging method is based on glucose metabolism evaluation in attractive lesions by measuring accumulated [^18^F]FDG, a glucose analog. Since glucose uptake is accelerated in cancer cells via anaerobic glycolysis, also known as the so-called “Warburg effect^10^, it is possible to detect tumors with high glucose metabolism^11^. Glucose metabolism including anaerobic glycolysis is reportedly influenced by glucose metabolic enzymes, such as glucose transporter-1 (GLUT1), pyruvate kinase-M2 (PKM2), pyruvate dehydrogenase-E1α (PDH-E1α), and carbonic anyhydrase-9 (CA9)^12–18^. GLUT1 is overexpressed in various cancers and is responsible for glucose uptake through the cell membrane^12–14^. PKM2 promotes anaerobic glycolysis, and its selective expression plays an important role in the Warburg effect^15^. The pyruvate dehydrogenase (PDH) complex, of which PDH-E1α plays a key role, catalyzes the conversion of pyruvate to acetyl coenzyme A (acetyl-CoA) and promotes aerobic glucose metabolism^16^. CA9 represents hypoxia inducible factor-1α, which stimulates glycolytic energy production by transactivating genes involved in extracellular glucose import and enzymes responsible for the glycolytic breakdown of intracellular glucose^17,18^. Therefore, these glucose metabolism enzyme levels might affect glucose uptake, including [^18^F]FDG, and might have a critical impact on the assessment of nodal stage using [^18^F]FDG-PET/CT.

In this study, we aimed to highlight the significance of glucose metabolism enzymes in the diagnosis of nodal stage by [^18^F]FDG-PET/CT and to define a new cut-off value for [^18^F]FDG-PET based on the expression of glucose metabolism enzymes.

## Results

### Relationship between clinical features and lymph node metastasis

The clinical features of all 223 patients based on lymph node metastasis are summarized in Table 1. No significant correlations were found between lymph node metastasis and age, sex, smoking history, or histology. In patients with lymph node metastasis, 54 patients had lymph node metastasis only in N1 area, 26 patients had only in N2 area, and 22 patients had both N1 and N2 area.

**Table 1:** Relationship between lymph node metastasis and clinical features in 223 patients with lung cancer.

Variables		Lymph node metastasis		
		Positive (N = 102)	Negative (N = 121)	*p* value
Age	< 65	28 (27.5%)	22 (18.2%)	
	> = 65	74 (72.5%)	99 (81.8%)	0.109
Sex	Female	33 (32.4%)	46 (38.0%)	
	Male	69 (67.6%)	75 (62.0%)	0.402
Smoking	Yes	79 (77.5%)	81 (66.9%)	
	No	23 (22.5%)	40 (33.1%)	0.101
Histology	Adenocarcinoma	67 (65.7%)	88 (72.7%)	
	Squamous cell carcinoma	25 (24.5%)	26 (21.5%)	
	Others^a^	10 (9.8%)	7 (5.8%)	0.426

^a^Others, adenosquamous cell carcinoma, large cell carcinoma, small cell carcinoma, pleomorphic carcinoma, and mucoepidermoid carcinoma.

### Expression of PDH-E1α, CA9, GLUT1, and PKM2 in primary lung cancers

**Figure 1:**
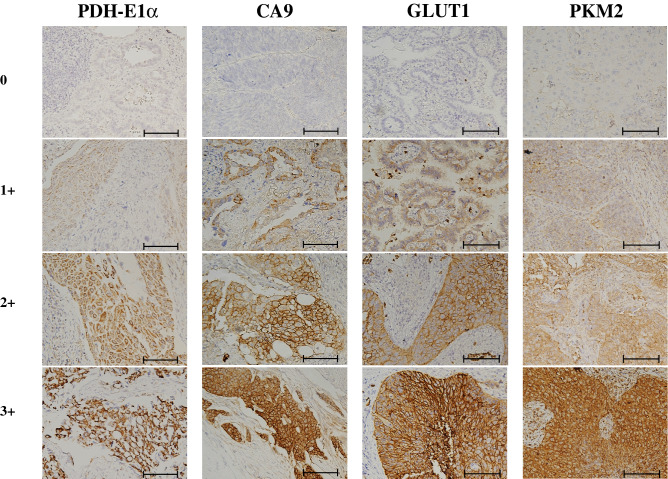
Immunostaining of PDH-E1α, CA9, GLUT1, and PKM2. The immunoreactivity of GLUT1, PKM2, PDH-E1α, and CA9 was evaluated according to the intensity staining of the cell membrane or cytoplasm of cancer cells. Immunostaining intensity score: 0, negative; 1+, weakly positive; 2+, positive; and 3+, strongly positive. Bar, 50 μm. PDH-E1α, pyruvate dehydrogenase E1α; CA9, carbonic anhydrase 9; GLUT1, glucose transporter-1, PKM2; pyruvate kinase-M2.

PDH-E1α and PKM2 were expressed in the cytoplasm, while CA-9 and GLUT1 were observed mainly in the cell membrane (Fig. 1). The immunoreactivity of GLUT1, PKM2, PDH-E1α, and CA9 was evaluated according to the intensity and proportion of immunoreactive cells stained at the membrane or cytoplasm.

### SUVmax of the primary lesion and metastatic lymph node according to the expression level of glucose metabolism enzymes

**Figure 2:**
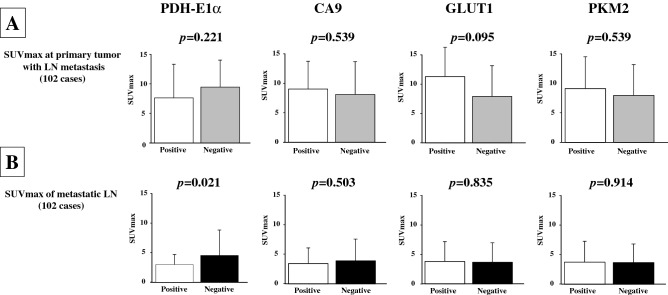
SUVmax of the primary lesion and metastatic lymph node according to the expression level of glucose metabolism enzymes. (**A**) Shows the SUVmax of primary tumors in 102 patients with lymph node (LN) metastasis. In contrast, there was no significant difference in the SUVmax of primary tumors according to the expression of glucose metabolism enzymes. (**B**) Shows the SUVmax of metastatic lymph nodes in all 102 patients. There is a significant difference in the SUVmax of metastatic lymph nodes according to the PDH-E1α expression, but not in GLUT1, CA9, and PKM2 expression. SUV, standard uptake value; PDH-E1α, pyruvate dehydrogenase E1α; CA9, carbonic anhydrase 9; GLUT1, glucose transporter-1, PKM2; pyruvate kinase-M2.

Figure 2A shows the SUVmax of the primary tumor in 102 patients with lymph node metastasis. There was no significant difference in the SUVmax of the primary tumor according to the expression levels of the glucose metabolism enzymes GLUT1, CA9, and PKM2. Figure 2B shows the SUVmax of the metastatic lymph nodes of all 102 patients. A significant difference in the SUVmax of metastatic lymph nodes was found according to PDH-E1α expression (*p* = 0.021), but not according to GLUT1, CA9, or PKM2 expression. There was no significant difference in the size of metastatic lymph node according to PDH-E1α expression in primary tumor (Supplement Fig. S2).

### New [^18^F]FDG-PET/CT cut-off value for lymph node metastasis based on PDH-E1α expression

**Figure 3:**
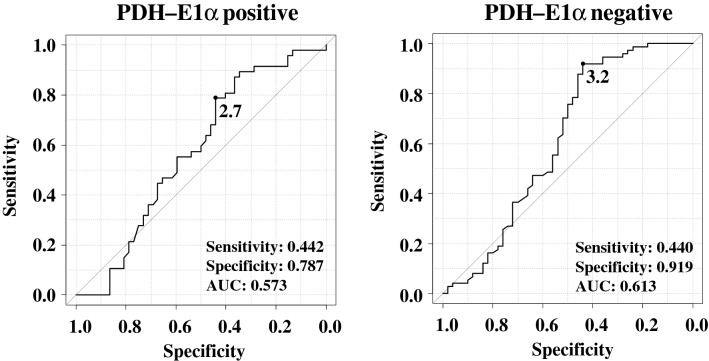
ROC curve of SUVmax according to PDH-E1α expression. In patients with PDH-E1α-positive primary tumors, the optimum cut-off value of SUVmax was 2.7 and AUC was 0.573. In contrast, in patients with PDH-E1α-negative expression tumors, the optimum cut-off value of SUVmax was 3.2 and AUC was 0.613. SUV, standard uptake value; PDH-E1α, pyruvate dehydrogenase E1α; ROC, receiver operating characteristic; AUC, area under the curve.

Since only PDH-E1α expression in the primary tumor showed a significant difference in SUVmax in metastatic lymph nodes, the receiver operating characteristic (ROC) curve of SUVmax according to PDH-E1α expression was constructed in Fig. 3. This was used to determine the new cut-off value of [^18^F]FDG-PET/CT for lymph node metastasis with the highest sensitivity and specificity with respect to distinguishing metastatic from non-metastatic lymph nodes. The optimum cut-off value of SUVmax was 2.7, and the area under the curve (AUC) was 0.573 in patients with PDH-E1α-positive primary tumors. In contrast, the optimum cut-off value of SUVmax was 3.2, and the AUC was 0.613 in patients with PDH-E1α-negative expression.

### Relationship between clinicopathological features and [^18^F]FDG-PET outcomes in 102 patients with lymph node metastasis

We compared the clinical features of the patients between the outcomes of the classical [^18^F]FDG-PET/CT and the new criteria according to PDH-E1α expression in the primary lesion (Table 2). There was a significant difference in age at an SUVmax cut-off of 2.5. In contrast, significant differences were found in age and smoking history at an SUVmax cut-off of 2.7 with PDH-E1α-positive and an SUVmax cut-off of 3.2 with PDH-E1α-negative expression.

**Table 2:** Relationship between [^18^F]FDG-PET/CT and clinicopathological features in 102 patients with lung cancer and lymph node metastasis.

Variables		[^18^F]FDG-PET/CT (Cut-off ≥ 2.5)			[^18^F]FDG-PET/CT (Cut-off: PDH-positive ≥ 2.7 PDH-negative ≥ 3.2)		
		Positive (n = 54)	Negative (n = 48)	*p* value	Positive (n = 54)	Negative (n = 48)	p value
Age	< 65	9 (16.7%)	19 (39.6%)		7 (15.6%)	21 (36.8%)	
	≥ 65	45 (83.3%)	29 (60.4%)	0.014	38 (84.4%)	36 (63.2%)	0.025
Sex	Female	14 (25.9%)	19 (39.6%)		12 (26.7%)	21 (36.8%)	
	Male	40 (74.1%)	29 (60.4%)	0.203	33 (73.3%)	36 (63.2%)	0.295
Smoking	Yes	46 (85.2%)	33 (68.8%)		40 (88.9%)	39 (68.4%)	
	No	8 (14.8%)	15 (31.2%)	0.059	5 (11.1%)	18 (31.6%)	0.017
Histology	Adenocarcinoma	35 (64.8%)	32 (66.7%)		30 (66.7%)	37 (64.9%)	
	Squamous cell carcinoma	15 (27.8%)	10 (20.8%)	0.59	12 (26.7%)	13 (22.8%)	0.679
	Others	4 (7.4%)	6 (12.5%)		3 (6.6%)	7 (12.3%)	
Lymphatic invasion	Negative	27 (50.0%)	17 (35.4%)		22 (48.9%)	22 (38.6%)	
	Positive	27 (50.0%)	31 (64.6%)	0.164	23 (51.1%)	35 (61.4%)	0.321
Venous invasion	Negative	25 (46.3%)	27 (56.2%)		21 (46.7%)	31 (46.7%)	
	Positive	29 (53.7%)	21 (43.8%)	0.33	24 (53.3%)	26 (53.3%)	0.55
Pleural invasion	Negative	31 (57.4%)	25 (52.1%)		28 (62.2%)	28 (49.1%)	
	Positive	23 (42.6%)	23 (47.9%)	0.691	17 (37.8%)	29 (50.9%)	0.231
Depth of invasion	T1 or T2	44 (81.5%)	35 (72.9%)		37 (82.2%)	42 (73.7%)	
	T3 or T4	10 (18.5%)	13 (27.1%)	0.348	8 (17.8%)	15 (26.3%)	0.348
CA9 expression	Negative	32 (59.3%)	30 (62.5%)		28 (62.2%)	34 (59.6%)	
GLUT1 expression	Positive	22 (40.7%)	18 (37.5%)	0.84	17 (37.8%)	23 (40.4%)	0.84
	Negative	34 (63.0%)	33 (68.8%)		31 (68.9%)	36 (63.2%)	
PKM2 expression	Positive	20 (37.0%)	15 (31.2%)	0.676	14 (31.1%)	21 (36.8%)	0.675
	Negative	32 (59.3%)	31 (64.6%)		28 (62.2%)	35 (61.4%)	
PDH expression	Positive	22 (40.7%)	17 (35.4%)	0.684	17 (37.8%)	22 (38.6%)	1
	Negative	27 (50.0%)	23 (47.9%)		22 (48.9%)	28 (49.1%)	
	Positive	27 (50.0%)	25 (52.1%)	0.846	23 (51.1%)	29 (50.9%)	1

[^18^F]FDG-PET/CT, [^18^F]fluorodeoxyglucose positron emission tomography/computed tomography; PDH–E1α, pyruvate dehydrogenase E1α; CA9, carbonic anhydrase 9; GLUT1, glucose transporter-1, PKM2; pyruvate kinase-M2.

### Comparison of sensitivity, specificity, positive predictive value (PPV), negative predictive value (NPV), positive likelihood ratio, and negative likelihood ratio between the classical and new criteria [^18^F]FDG-PET outcome

The ROC curve indicated that the [^18^F]FDG-PET/CT cut-off value for lymph node metastasis might be adequate at 2.7 and 3.2 in patients with PDH-E1α-positive and -negative primary tumors, respectively (Fig. 3). We then compared the true and false positive and negative cases of [^18^F]FDG-PET/CT in the diagnosis of lymph node metastasis between the classical and new criteria [^18^F]FDG-PET/CT outcomes in Table 3. When the cut-off value of SUVmax was set to 2.5, the sensitivity and specificity were 0.529 and 0.562, respectively, the PPV and NPV were 0.505 and 0.586, respectively, and the positive and negative likelihood ratios were 1.208 and 0.838, respectively. On the other hand, when the cut-off value of SUVmax was set according to PDH-E1α expression, the sensitivity and specificity were 0.441 and 0.868, respectively, the PPV and NPV were 0.738 and 0.648, respectively, and the positive and negative likelihood ratios were 3.341 and 0.644, respectively.

**Table 3:** Comparison of sensitivity, specificity, predictive value, and likelihood ratio of [^18^F]FDG-PET/CT between the conventional SUVmax cut-off and PDH-followed SUVmax cut-off.

	[^18^F]FDG-PET/CT SUVmax cut-off value					
	> 2.5			PDH-positive cases ≥ 2.7 PDH-negative cases ≥ 3.2		
Lymph node metastasis	Positive	Negative	Total	Positive	Negative	Total
Positive	54	48	102	45	57	102
Negative	53	68	121	16	105	121
	107	116	223	61	162	223

	SUVmax cut-off value					
Variables	> 2.5	PDH-positive cases ≥ 2.7 PDH-negative cases ≥ 3.2				
Sensitivity	0.529	0.441				
Specificity	0.562	0.868				
Positive predictive value	0.505	0.738				
Negative predictive value	0.586	0.648				
Positive likelihood ratio	1.208	3.341				
Negative likelihood ratio	0.838	0.644				

[^18^F]FDG-PET/CT, [^18^F]fluorodeoxyglucose positron emission tomography/computed tomography; SUVmax, maximum standardized uptake value; PDH, pyruvate dehydrogenase.

SUVmax, maximum standardized uptake value; PDH, pyruvate dehydrogenase.

## Discussion

Preoperative nodal stage is important for determining the surgical procedure for patients with lung cancer^2,3,8^. In [^18^F]FDG-PET false-negative cases with metastatic lymph nodes, surgical resection may be performed despite chemotherapy being the first choice of treatment. In this study, we proposed a new [^18^F]FDG-PET/CT cut-off value for lymph node metastasis in lung cancer with respect to PDH-E1α expression in primary tumors.

[^18^F]FDG accumulation in primary lung cancer has been shown to be influenced by several enzymes, including glucose metabolism enzymes^7,9,19^; therefore, it may be better to set a cut-off value in relation to each enzyme. The SUVmax value in metastatic lymph nodes was significantly different between PDH-E1α-positive and -negative tumors, while no significant difference in SUVmax was found based on the expression levels of the other glucose metabolism enzymes GLUT1, PKM2, and CA9. The SUVmax cut-off value was re-evaluated using the ROC curve according to PDH-E1α expression in the primary tumor. ROC curve analysis indicated that the [^18^F]FDG-PET/CT cut-off value for lymph node metastasis might be adequate at 2.7 in patients with PDH-E1α-positive primary tumors and 3.2 in patients with PDH-E1α-negative tumors. The SUVmax cut-off value of [^18^F]FDG-PET/CT to diagnose lymph node metastasis conventionally been 2.5^20–22^. Comparatively, our new [^18^F]FDG-PET/CT cut-off value improved the specificity and NPV from 0.562 to 0.868 and from 0.586 to 0.648, respectively. These findings suggest that our new PET cut-off value based on the PDH-E1α expression is more specific. This can reduce the number of false-negative cases in the preoperative nodal stage, thereby reducing the incidence of postoperative lymph node recurrence.

A systematic review reported that [^18^F]FDG-PET/CT has a sensitivity of 0.774 and specificity of 0.901 in diagnosing preoperative lymph node metastasis^23^. Although there were many studies aimed to improve the accuracy of [^18^F]FDG-PET/CT in diagnosing lymph node metastasis, most studies reported less accuracy in clinical practice^5,21,24–26^. On the other hand, our new SUVmax cut-off value according to PDH-E1α expression showed higher specificity for [^18^F]FDG-PET in the diagnosis of lymph node metastasis. To improve the accuracy of the test, new original standards have been reported, such as changing the cut-off value or adding further parameters in addition to SUVmax^5,21,24–26^, but they are based on clinical parameters only. The novel aspect of our study is to define a new SUVmax cut-off value for [^18^F]FDG-PET/CT based on the molecular biological aspects of glucose metabolic enzymes. PDH-E1α catalyzes the conversion of pyruvate to acetyl-CoA, which enters the tricarboxylic acid cycle via aerobic glycolysis^27^. A lack of PDH promotes anaerobic glycolysis and an increase in glucose consumption, resulting in the stimulation of [^18^F]FDG uptake, which is associated with a high SUVmax value in cancer cells. Since the molecular biology of cancer cells might not be very different between the primary lesion and metastatic lymph nodes^28–31^, SUVmax in metastatic lymph nodes might be high in cases with PDH-E1α-negative primary tumors. These findings suggest that the examination of PDH-E1α expression using a preoperative biopsy specimen may be useful to determine the hilar nodal stage before surgery using [^18^F]FDG-PET/CT under a new SUVmax cut-off value.

In this study, we did not examine the expression of glucose metabolism enzymes in metastatic lymph nodes, but the expression of PDH-E1α, GLUT1, PKM2, and CA9 between primary and metastatic lymph nodes may differ. In addition, the microenvironment surrounding cancer cells differs between primary tumors and metastatic lymph nodes^32,33^. Therefore, if the expression of these enzymes is acquired and regulated by the microenvironment, this may explain the different effects of PDH-E1α and other enzymes on metastatic lymph nodes.

This study has three limitations. First, this was a retrospective study that analyzed [^18^F]FDG-PET/CT imaging and clinicopathological data. A prospective study of PDH-E1α expression using biopsy specimens will be necessary to determine the nodal stage before surgery. Although a significant difference in the SUVmax of metastatic lymph nodes was found in accordance with the PDH-E1α expression of primary tumor, the preoperative lymph node status is necessary for determining the appropriate treatment strategy in clinical practice. Therefore, it may be necessary to investigate the SUVmax of lymph nodes and glucose metabolic enzymes using the preoperative biopsy specimens, rather than surgical specimens. Second, we evaluate only SUVmax among the data that could be obtained from FGD-PET imaging. It will be more reliable by using the combinations of other parameters such as SUVpeak, total lesion glycolysis, metabolic tumor volume. However, these parameters are not measured in our institute and it was difficult to analyze them in this study. Collection of those data and further investigations are ongoing. Third, the number of patients was limited; thus, it was not possible to analyze all factors in all patients.

In conclusion, the SUVmax cut-off value for metastatic lymph nodes depends on the PDH-E1α expression level in primary lung cancer. The new SUVmax cut-off value according to PDH-E1α expression shows higher specificity of [^18^F]FDG-PET for diagnosing lymph node metastasis and is useful for predicting accurate nodal stage before surgery.

## Methods

### Patient selection and ethical statement

A clinicopathological record of 632 patients with lung cancer who underwent surgical resection at Osaka City University between January 2012 and September 2019 were retrospectively reviewed. A total of 223 patients with lung cancer who underwent [^18^F]FDG-PET/CT within 2 months before surgery were analyzed in this study. We excluded patients with lack of lymph node dissection, preoperative [^18^F]FDG-PET/CT, and adequate [^18^F]FDG-PET/CT data (including examined in other hospital and mismatch of inspection device). A diagram of the process by which the cases were selected for this study is shown in Supplement Fig. S1. All the patients were pathologically diagnosed with primary lung cancer and its nodal status was determined via surgical specimens. Clinicopathological background was retrospectively investigated. Of the 223 patients with lung cancer, 102 had pathologically diagnosed lymph node metastasis, and 121 showed pathologically no lymph node metastasis. Pathological findings were determined according to the 8th edition of the Union for International Cancer Control TNM classification. This study was conducted in accordance with the principles of the Declaration of Helsinki and approved by the Osaka City University Ethics Committee (approval number: 2019-006). Informed consent was obtained from all the patients, and all methods were performed in accordance with relevant guidelines and regulations.

### [^18^F]FDG-PET/CT imaging

All patients underwent preoperative [^18^F]FDG-PET/CT scanning using a Biograph 16 scanner (Siemens Medical Solutions, Erlangen, Germany). The Biograph 16 consists of a 16-slice CT detector and a Lu2SiO5[Ce] crystal block, which were periodically inspected four times a year. The PET scanner field of view had an 830-mm ring diameter and a 162-mm axial length. The patient was fasting for 6 h prior to the examination^34^. The injected activity was 3 MBq/kg of [^18^F]FDG, and PET scans were acquired from the parietal to the proximal thigh at 2 min on bed position, 60 min after injection. PET images were reconstructed using iterative reconstruction (iterations: 2, subsets: 8). Noise reduction was performed by smoothing the images with a Gaussian filter (full width at half maximum, 5 mm). Whole-body CT scans were acquired with patients in the supine position, from the parietal to the proximal thigh (120 kVp; 100 mA in auto mA mode). All data were subjected to attenuation correction based on CT data. The scatter correction followed a single-scatter simulation method.

### SUVmax measurement

The SUVmax of [^18^F]FDG-PET/CT at the hilar lymph node was measured in the axial image slice with the highest [^18^F]FDG activity concentration. The SUVmax was calculated as follows: SUVmax = maximum radioactivity concentration in tissue (Bq/g)/[injected dose (Bq)/patient's body weight (g)]. For patients with multiple lymph node metastases, the SUVmax of the largest lymph node was used for statistical analysis. If lymph node metastases were present in both N1 (ipsilateral peribronchial, hilar, or intrapulmonary lymph nodes) and N2 areas (ipsilateral mediastinal or subcarinal lymph nodes), the SUVmax of the N2 lymph node was used.

### Immunostaining

Paraffin-embedded sections from primary tumor of 102 patients with lymph node metastasis were deparaffinized in xylene and hydrated in decreasing concentrations of ethyl alcohol. The sections were incubated with 3% hydrogen peroxide to block endogenous peroxidase activity, and heated for 10 min at 105 °C by autoclave in Target Retrieval Solution (DAKO, Carpinteria, CA, USA). Nonspecific binding was blocked by incubating with 10% normal rabbit serum for 10 min. The specimens were incubated with anti-GLUT1 antibodies (sc-377228; 1:150; Santa Cruz, Dallas, TX, USA; RRID: AB_2716767) for 30 min at room temperature, anti-PKM2 antibodies (sc-365684; 1:200; Santa Cruz; RRID: AB_10844484) at 4 °C overnight, anti-PDH-E1α antibodies (sc-377092; 1:100; Santa Cruz; RRID: AB_2716767)^35^ at 4 °C overnight, and with CA9 antibodies (NB100–417; 1:1000; Novus Biologicals, Centennial, CO, USA; RRID: AB_10003398) for 30 min at room temperature. These sections were incubated with a mouse linker for 10 min and peroxidase-labeled polymer (Histofine SAB-PO(M) kit, Nichirei Biosciences Inc., Tokyo, Japan) for 5 min, followed by counterstaining with Mayer’s hematoxylin.

### Immunohistochemical determination

The immunoreactivity of GLUT1, PKM2, PDH-E1α, and CA9 was evaluated based on the intensity of membranous staining at the deepest level of the tumor and the proportion of immunoreactive cells. The immunostaining intensity score was rated as follows: 0, negative; 1+, weakly positive; 2+, positive; and 3+, strongly positive. The immunostaining proportion score was calculated as an estimate of the proportion of positive cells: 0, no immunoreactive cells; 1+, < 30% immunoreactive cells; 2+, 40–70% immunoreactive cells; and 3+, > 80% immunoreactive cells. The total score was calculated as the summation of the immunostaining intensity score and proportion score, ranging from 0 to 6, and the expression of all antibodies was considered positive when the summation score was ≥ 4.

### Statistical analysis

Differences in clinicopathological factors were assessed using t-test, χ^2^ test, and Fisher’s exact test. The ROC curve obtained by threshold method was used to determine the SUVmax cut-off value by using the SUVmax value of lymph node and its presence of metastasis. The optimal cut-off values were established as the ones that that gave the highest result for the sum of sensitivity and specificity. In all tests, a *p*-value of < 0.05 was considered statistically significant. All statistical analyses were performed using EZR (Saitama Medical Center, Jichi Medical University, Saitama, Japan), which is a graphical user interface of R and a modified version of the R commander (The R Foundation for Statistical Computing, Vienna, Austria)^36^. The sensitivity, specificity, PPV, NPV, and accuracy for lymph node metastasis were calculated using either the conventional criterion (SUVmax cut-off of 2.5) or the modified criterion (a new cut-off of SUVmax according to PDH expression) by creating new 2 × 2 tables.

## Supplementary Information

Supplementary Figure S1.

Supplementary Figure S2.

## Data Availability

The datasets used and analyzed during the current study available from the corresponding author on reasonable request.
