# Supplementary material for: Usefulness of pyruvate dehydrogenase-E1α expression to determine SUVmax cut-off value of [18F]FDG-PET for predicting lymph node metastasis in lung cancer
Source: Sci Rep. 2023 Jan 28;13:1565. doi: 10.1038/s41598-023-28805-8 (PMC9884208; doi:10.1038/s41598-023-28805-8)
Supplement: Supplementary file 1 — Supplementary Figure S1. [file 41598_2023_28805_MOESM1_ESM.pptx]

## Slide 1
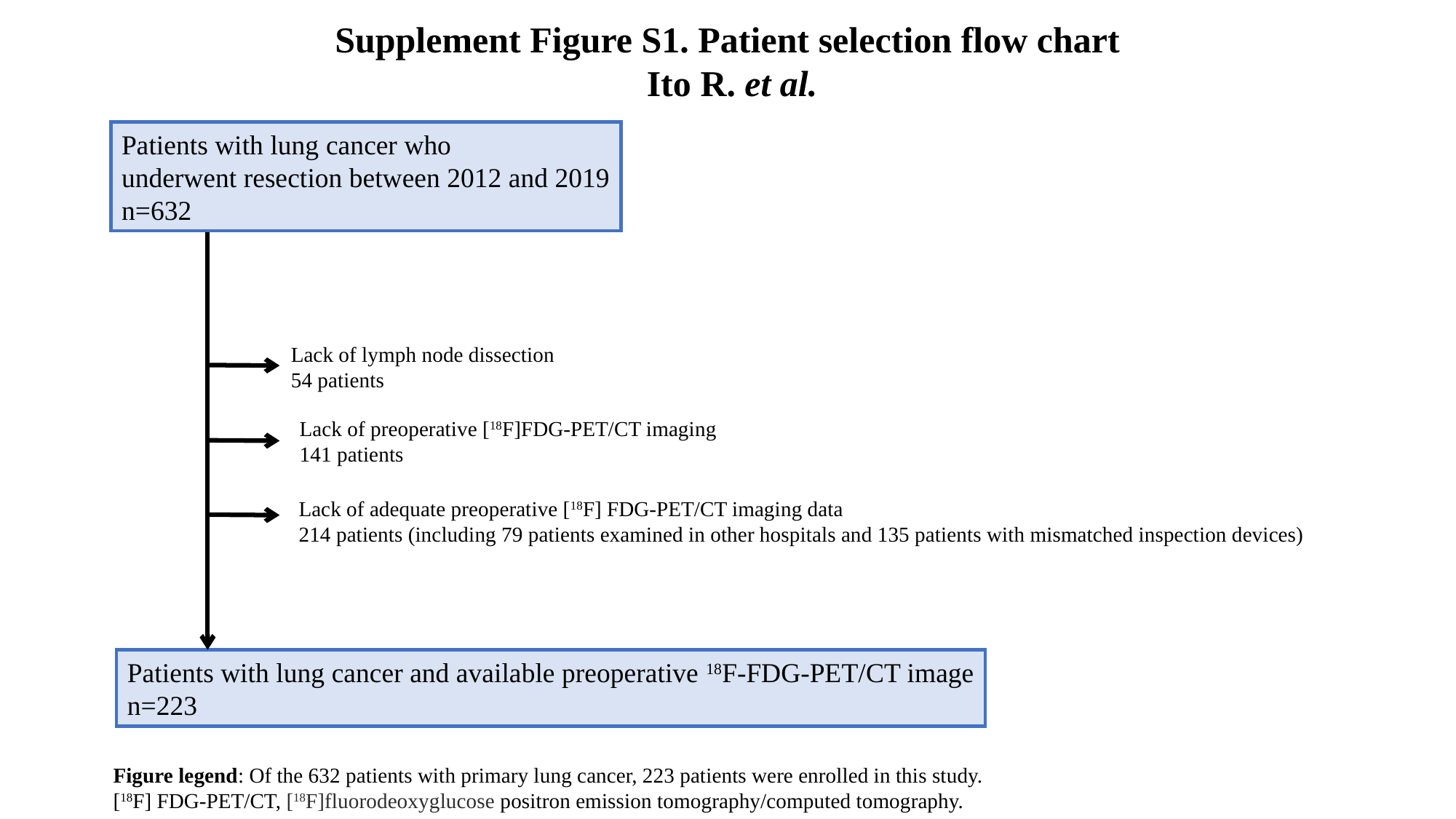

Supplement Figure S1. Patient selection flow chart
 Ito R. et al.
Patients with lung cancer who
underwent resection between 2012 and 2019
n=632
Lack of lymph node dissection
54 patients
Lack of preoperative [18F]FDG-PET/CT imaging
141 patients
Lack of adequate preoperative [18F] FDG-PET/CT imaging data
214 patients (including 79 patients examined in other hospitals and 135 patients with mismatched inspection devices)
Patients with lung cancer and available preoperative 18F-FDG-PET/CT image
n=223
Figure legend: Of the 632 patients with primary lung cancer, 223 patients were enrolled in this study.
[18F] FDG-PET/CT, [18F]fluorodeoxyglucose positron emission tomography/computed tomography.
