# Supplementary material for: Usefulness of pyruvate dehydrogenase-E1α expression to determine SUVmax cut-off value of [18F]FDG-PET for predicting lymph node metastasis in lung cancer
Source: Sci Rep. 2023 Jan 28;13:1565. doi: 10.1038/s41598-023-28805-8 (PMC9884208; doi:10.1038/s41598-023-28805-8)
Supplement: Supplementary file 2 — Supplementary Figure S2. [file 41598_2023_28805_MOESM2_ESM.pptx]

## Slide 1
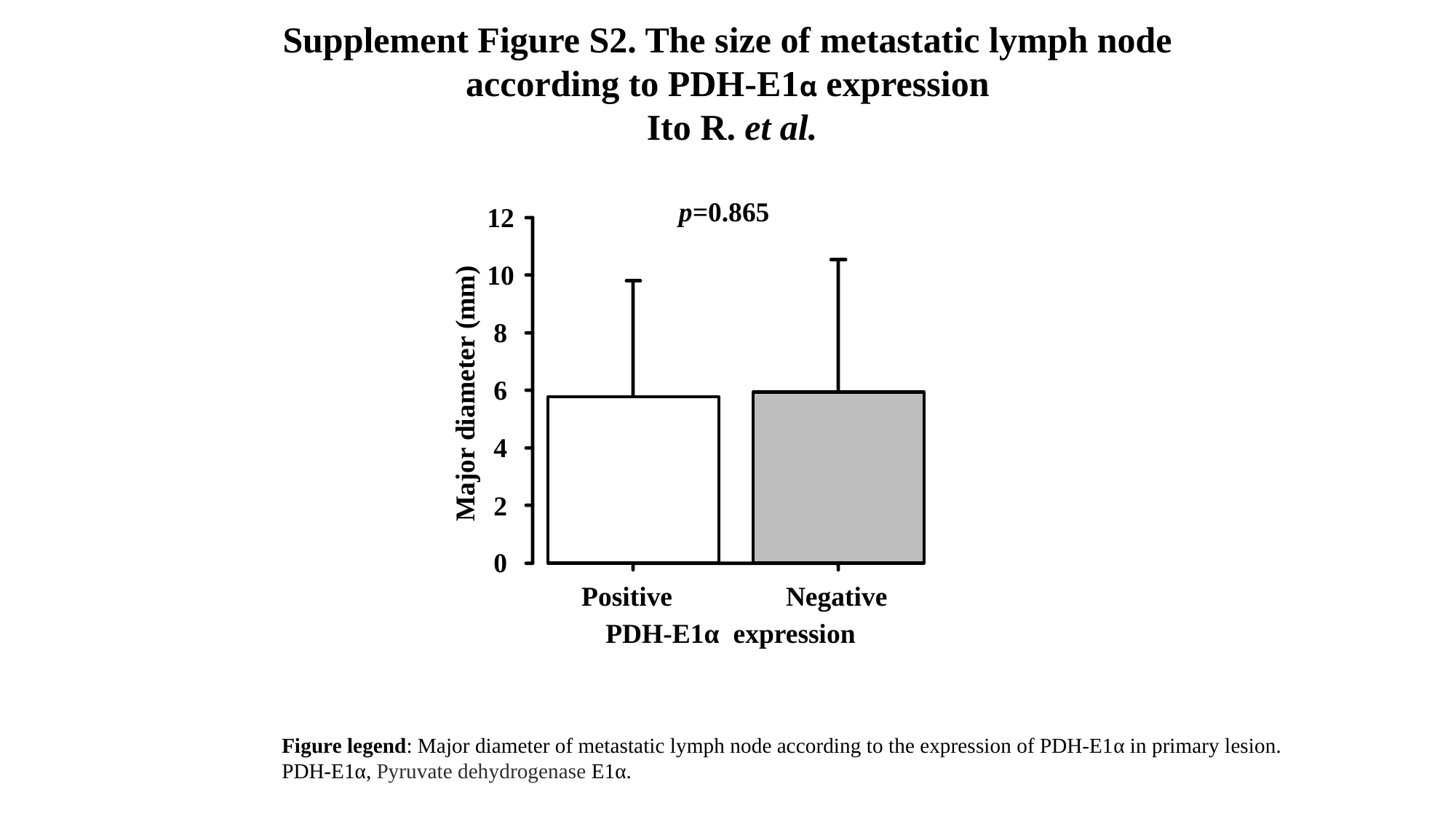

Supplement Figure S2. The size of metastatic lymph node according to PDH-E1α expression
 Ito R. et al.
p=0.865
12
10
8
Major diameter (mm)
6
4
2
0
Positive
Negative
PDH-E1α expression
Figure legend: Major diameter of metastatic lymph node according to the expression of PDH-E1α in primary lesion.
PDH-E1α, Pyruvate dehydrogenase E1α.
